# Partial Oral Antibiotic Therapy for Infective Endocarditis: A Practical Review of Current Evidence and Guidelines

**DOI:** 10.3390/pathogens15090924

**Published:** 2026-09-02

**Authors:** Thomas Roland, Jean Cyr Yombi

**Affiliations:** 1 Department of Internal Medicine and Infectious Diseases, CHU HELORA (Centre Hospitalier Universitaire Helora), 7000 Mons, Belgium; thomas.roland@helora.be; 2 Department of Internal Medicine and Infectious Diseases, Cliniques Universitaires Saint-Luc, 1200 Brussels, Belgium

**Keywords:** infective endocarditis, oral antibiotics, partial oral antibiotic therapy (POAT), POET trial, step-down therapy, antibiotic stewardship

## Abstract

Infective endocarditis (IE) is associated with an in-hospital mortality rate of 15–30%. Standard treatment requires 2 to 6 weeks of intravenous (IV) antibiotic therapy, resulting in prolonged hospitalization with its attendant risks, including catheter-related complications, increased length of stay, and cost. This review summarizes the current evidence, eligibility criteria, antibiotic regimen selection, and practical aspects of partial oral antibiotic therapy (POAT) for IE. Oral treatment is already recommended for right-sided *Staphylococcus aureus* IE and IE caused by selected atypical pathogens (e.g., *Brucella* spp., *Coxiella burnetii*, *Bartonella* spp., and *Tropheryma whipplei*). The landmark Partial Oral Treatment of Endocarditis trial (2019) demonstrated the non-inferiority of POAT—initiated after at least 10 days of IV therapy—versus continued IV treatment for left-sided IE caused by streptococci, *Enterococcus faecalis*, *S. aureus*, or coagulase-negative staphylococci. Long-term follow-up at 5.4 years demonstrated lower all-cause mortality in the POAT group. The 2023 European Society of Cardiology Guidelines now formally endorse POAT for selected patients fulfilling strict criteria. Despite robust evidence, POAT is currently implemented in fewer than half of eligible patients. These findings have since been corroborated by the WikiGuidelines Group consensus statement and the French Société de Pathologie Infectieuse de Langue Française–Association pour l’Étude et la Prévention de l’Endocardite Infectieuse position statement, and extended by real-world evidence (the ENDO-ORAL study) suggesting that carefully selected patients falling outside strict trial-based eligibility criteria may also benefit from oral step-down therapy. In appropriately selected and clinically stable patients, POAT is supported by randomized and real-world evidence as an alternative to prolonged IV therapy. Strict adherence to eligibility criteria, appropriate antibiotic selection based on pathogen susceptibility, and close clinical follow-up are essential to optimize outcomes.

## 1. Introduction

Current guidelines for the management of infective endocarditis (IE) recommend 2 to 6 weeks of intravenous (IV) antibiotic therapy, depending on the causative microorganism and the presence of a native or prosthetic valve [1,2]. This requirement frequently results in prolonged hospitalization, cost, and catheter-related complications. Recent evidence has highlighted the feasibility of switching to oral antibiotic therapy after initial IV stabilization, a paradigm already established in other conditions requiring prolonged antimicrobial treatment, such as bone and joint infections [3,4].

Maintaining a central venous catheter throughout the treatment course is associated with a nearly threefold increase in the risk of catheter-related bloodstream infections [5] and prolonged hospitalization itself is independently associated with adverse outcomes, including functional decline in elderly patients, post-traumatic stress disorder, and healthcare-associated complications [6,7]. Outpatient parenteral antimicrobial therapy and partial oral antibiotic therapy (POAT) for IE may substantially reduce hospital length of stay and related complications [8,9,10]. Nevertheless, in routine clinical practice, oral step-down therapy is implemented in fewer than half of eligible patients [11] despite evidence suggesting a reduction in mortality. Rather than providing another summary of efficacy data alone, this practical review integrates trial and real-world evidence with contemporary guideline and position-statement recommendations, and translates them into a bedside-oriented framework for patient selection, oral regimen choice, monitoring, and areas of remaining uncertainty.

Search strategy and scope: A non-systematic literature search of PubMed/MEDLINE was performed for English-language publications on oral and partial oral antibiotic therapy for infective endocarditis, covering January 2000 to June 2026, using combinations of the terms “infective endocarditis”, “oral antibiotic”, “oral step-down”, and “partial oral treatment”, together with the names of the major trials, cohorts, and guideline bodies discussed herein (e.g., Partial Oral Treatment of Endocarditis (POET) trial, European Society of Cardiology (ESC), WikiGuidelines, Société de Pathologie Infectieuse de Langue Française (SPILF)–Association pour l’Étude et la Prévention de l’Endocardite Infectieuse (AEPEI)). Reference lists of retrieved articles and recent guideline documents were hand-searched for additional relevant studies. Given the narrative, expert-opinion format of this review, no formal risk-of-bias assessment or systematic study-selection protocol (e.g., PRISMA) was applied; articles were selected for inclusion based on clinical relevance and methodological quality as judged by the authors. This review focuses on adult patients with native or prosthetic valve IE; the management of infective endocarditis in children is outside its scope.

## 2. Prognosis and Clinical Context

The in-hospital mortality rate of IE ranges from 15% to 30% [12]. Poor outcomes at admission are associated with patient comorbidities, the presence of IE-related clinical complications, the causative microorganism, and echocardiographic findings (see supplementary file of Ref. [12]).

*Staphylococcus aureus* infection is a predictor of embolic events and short-term mortality, with mortality rates ranging from 25% to 47% [11,13,14]. Its adverse prognostic impact is consistent with the marked virulence of *S. aureus*, including efficient adhesion to damaged endothelium and prosthetic material, rapid tissue invasion and destruction, a high propensity for persistent bacteremia and embolization, and biofilm formation on intracardiac prosthetic (see Refs. [14,15] and material supplementary file of Ref. [12]). In contrast, factors associated with a favorable outcome include symptom duration exceeding one month before diagnosis, streptococcal infection (in-hospital mortality 4–9%), and cardiac surgery when indicated (see Ref. [13] and supplementary file of Ref. [12]). Notably, Chirio et al. demonstrated that iatrogenic factors—particularly healthcare-associated infections occurring during prolonged hospitalization—are independently associated with an unfavorable prognosis [16]. This finding underscores the importance of strategies aimed at reducing the duration of in-patient care.

Beyond individual clinical outcomes, quality of life is substantially affected in IE survivors. Verhagen et al. reported significant rates of health-related quality of life impairment and post-traumatic stress disorder among survivors of left-sided native valve IE [6], further emphasizing the burden associated with prolonged hospitalization.

## 3. General Principles of Treatment

IV antibiotic therapy ensures complete bioavailability and rapid attainment of peak serum concentrations, which is particularly advantageous in critically ill patients. In contrast, oral antibiotic therapy is associated with more variable gastrointestinal absorption and systemic bioavailability [3].

Historically, effective treatment of IE has relied on prolonged IV antibiotic therapy. This paradigm emerged during the penicillin era, when poor oral bioavailability necessitated parenteral administration. Furthermore, bacteria embedded within cardiac vegetations are relatively inaccessible to antimicrobial agents and the host immune response. Cardiac vegetations create a high-density bacterial inoculum that reduces the efficacy of certain antibiotics—the so-called “inoculum effect”—and constitute a structural barrier to drug penetration [3,17].

The choice and duration of antimicrobial therapy in IE depend on pathogen identification, antimicrobial susceptibility testing, the presence of prosthetic material (prosthetic valves or cardiac implantable electronic devices (CIED)), and local or systemic complications such as valvular abscesses or embolic events. These factors collectively necessitate an individualized approach to treatment. Antimicrobial resistance is a major concern in *S. aureus* IE. Methicillin resistance markedly restricts therapeutic options by limiting the use of preferred antistaphylococcal beta-lactams, while reduced susceptibility or resistance to other key agents may further complicate treatment. Antimicrobial therapy should therefore remain guided by susceptibility testing and ensure adequate drug exposure [2,12].

The 2015 guidelines of the ESC and the American Heart Association (AHA) acknowledged outpatient parenteral antimicrobial therapy or, in selected cases, oral step-down therapy, although supporting evidence remained limited at that time [1,2,18,19].

## 4. Early Evidence for Oral Treatment of Infective Endocarditis

IE caused by atypical pathogens—including *Brucella* spp., *Coxiella burnetii*, *Bartonella* spp., *Legionella* spp., *Mycoplasma* spp., and *Tropheryma whipplei*—is already routinely treated with oral antibiotic regimens incorporating doxycycline, TMP-SMX, rifampicin, levofloxacin, clarithromycin or hydroxychloroquine [1,2,12,20].

The earliest trials of oral IE treatment following the introduction of sulfonamides, tetracyclines, and macrolides yielded disappointing results [17]. Nevertheless, a case report published in 1978 described the successful oral treatment of prosthetic valve *Enterococcus faecalis* IE in a patient with severe peripheral thrombophlebitis precluding prolonged IV access [21]. Heldman et al. demonstrated that an oral regimen combining ciprofloxacin and rifampicin could serve as effective step-down therapy for right-sided *S. aureus* IE in injection drug users [22]. Birmingham et al. and Falagas et al. subsequently explored linezolid—an oxazolidinone with near-complete oral bioavailability—for *E. faecium* IE. However, its use may be limited by hematological toxicity (anemia, thrombocytopenia), peripheral neuropathy, and serotonin syndrome risk in patients co-prescribed selective serotonin reuptake inhibitors [23,24].

Tissot et al. demonstrated that early oral switch to high-dose trimetoprim-sulfametoxazol (TMP-SMX) (960/4800 mg/day) combined with clindamycin was a safe alternative for the treatment of methicillin-susceptible *S. aureus* IE, with outcomes comparable to IV therapy [25]. The 2015 ESC guidelines subsequently endorsed this oral regimen as an option for uncomplicated right-sided native-valve IE caused by methicillin-susceptible *S. aureus (MSSA)* [1].

In a randomized study involving 30 patients, Stamboulian et al. demonstrated that oral amoxicillin step-down therapy was both effective and safe following two weeks of IV or intramuscular ceftriaxone for uncomplicated streptococcal IE caused by isolates with a penicillin minimum inhibitory concentration (MIC) below 0.12 μg/mL. Patients with indications for surgery, significant valvular regurgitation, abscesses, embolic events, or prosthetic valves were excluded [26].

A retrospective study published by Demonchy et al. in 2011 found no difference in mortality between patients receiving oral step-down therapy with a fluoroquinolone-rifampicin combination and those receiving continued intravenous therapy [27]. In 2016, Mzabi et al. conducted a retrospective study demonstrating that, in staphylococcal and streptococcal IE, oral step-down therapy using combinations of amoxicillin, clindamycin, linezolid, fluoroquinolones, or rifampicin was non-inferior to continued IV therapy with respect to relapse and mortality rates [28]. The AHA currently recognizes linezolid as an alternative for *E. faecium* IE and fluoroquinolones as alternatives to ceftriaxone for HACEK organism IE [2].

## 5. Principles and Advantages of Partial Oral Antibiotic Therapy

The success of antimicrobial therapy depends on three key factors: the susceptibility of the microorganism to the selected agent, the achievement of adequate antibiotic concentrations at the site of infection, and an appropriate duration of treatment [29]. Regardless of the route of administration, the critical determinant of efficacy is the attainment of sufficient antibiotic concentrations within the vegetation. Several modern antimicrobial agents exhibit excellent oral bioavailability and achieve serum concentrations adequate to eradicate the bacteria responsible for IE.

POAT may prevent an estimated 10–60% of IV-related adverse events (including catheter-related bloodstream infection, thrombophlebitis, and line-associated complications), while reducing healthcare costs and the need for medication-related procedures [17]. In specific patient populations, such as people who inject drugs (PWID), maintaining venous access may be undesirable or impossible owing to poor vascular access [29]. Moreover, oral therapy can support antimicrobial stewardship by avoiding unnecessary prolonged IV administration and its associated devices and resource use, while reducing the environmental impact of IV formulations and disposables [30]. This should not be interpreted as evidence that POAT directly reduces antimicrobial resistance; stewardship still requires the narrowest effective spectrum, susceptibility-guided selection, appropriate dosing, and avoidance of unnecessary treatment prolongation.

Several prerequisites must be fulfilled before considering a switch to oral therapy, depending on both patient-specific factors and the causative microorganism. The patient must be clinically stable, with controlled bacteremia and evidence of infection response. Oral intake must be reliable, and no cognitive impairment or social factors compromising adherence should be present. The antimicrobial susceptibility profile of the pathogen must support the use of oral agents. Some drugs require close monitoring for toxicity—particularly high-dose rifampicin and linezolid. The use of two active oral agents is primarily intended to ensure adequate antimicrobial exposure at the site of infection and to minimize the risk of emergent resistance, rather than to exploit pharmacodynamic synergy [16].

## 6. Recent Evidence

### 6.1. The POET Trial

The landmark POET trial, published by Iversen et al. in 2019, was a multicenter, non-inferiority, randomized controlled trial involving 400 patients with left-sided IE caused by streptococci, *E. faecalis*, *S. aureus*, or coagulase-negative staphylococci [8] A total of 201 patients underwent a switch to oral antibiotic therapy after at least 10 days of IV treatment or at least 7 days after cardiac surgery. The primary composite endpoint—all-cause mortality, unplanned cardiac surgery, embolic events, or relapse of bacteremia within 6 months—occurred in 12.1% of patients in the oral group versus 12.6% in the IV group (between-group difference, −0.5 percentage points; 90% confidence interval, −6.2 to 5.2), meeting the prespecified non-inferiority margin of 10 percentage points.

Eligible patients were at least 18 years old, clinically stable, and receiving IV antibiotic therapy for IE. Randomization was performed after a favorable clinical response following at least 10 days of IV treatment or at least 7 days after cardiac surgery. Patients with a valvular abscess or severe valvular dysfunction requiring surgery at the time of randomization were excluded. Aortic valve involvement was the most common presentation, while prosthetic valve endocarditis accounted for 27% of cases (107 patients).

Oral antibiotic regimens were selected according to susceptibility testing and available clinical evidence [Table 1]. All regimens combined two agents from different antimicrobial classes with high oral bioavailability. Therapeutic drug monitoring was performed during treatment. Subtherapeutic serum concentrations were observed in seven patients (rifampicin in three, moxifloxacin in two, linezolid in one, and dicloxacillin in one); however, therapeutic concentrations of the companion antibiotic were maintained in all cases, and no treatment modifications were deemed necessary.

### 6.2. Long-Term Follow-Up Data

Long-term follow-up data from the POET trial further support the safety and durability of POAT. After a mean follow-up of 5.4 years, all-cause mortality was 23.4% in the POAT group versus 35.2% in the IV group (hazard ratio, 0.65; 95% CI, 0.44–0.96), a statistically significant difference [33]. The causes of death were similar in both groups, comprising cardiovascular disease, infections, and malignancies. It is noteworthy, however, that a degree of selection bias cannot be excluded, given that only clinically stable patients without indications for immediate surgery were eligible for randomization.

### 6.3. The Danish POETry Study

The Danish POETry study, which assessed real-world implementation of POAT following its endorsement by the 2019 POET trial, demonstrated that 72% of patients fulfilled all eligibility criteria for POAT [11]. Among the remaining 28% who did not meet all criteria, the composite adverse outcome rate—including all-cause mortality, unplanned cardiac surgery, embolic events, and recurrent bacteremia—reached 22%, compared with 9% among eligible patients. Six-month mortality was 16% in non-eligible patients versus 5% in those fulfilling all eligibility criteria. Hospital readmission due to intolerance of oral therapy was observed in approximately 6% of patients. These findings highlight the critical importance of strict adherence to patient selection criteria.

Despite these encouraging results, POAT was implemented in only 43% of eligible patients in routine clinical practice. The presence of a valvular abscess, *S. aureus* infection, or a CIED was independently associated with a lower likelihood of oral step-down therapy.

### 6.4. Real-World Evidence

More recently, Freling et al. conducted a multicenter retrospective study including 257 patients, of whom 46 received POAT and 211 completed IV treatment [34]. No significant differences were observed between groups regarding recurrent bacteremia or hospital readmission. However, adverse events were significantly less frequent in the POAT group, consistent with prior evidence indicating a favorable safety profile for oral step-down therapy.

Rallet et al. recently reported the ENDO-ORAL study, a French bicentric retrospective cohort of 333 patients with definite IE treated between 2016 and 2023, of whom 100 (30%) underwent an oral switch after a median of 16 days of effective IV treatment [35]. After propensity-score weighting to account for indication bias, treatment failure at 90 days did not differ significantly between the oral-switch and exclusive-IV groups (hazard ratio 0.55, 95% CI, 0.26–1.17), and results were consistent in a matched sensitivity analysis (hazard ratio 0.65, 95% CI, 0.30–1.41). Patients who switched to oral therapy had significantly more days alive and out of hospital than those who remained on IV therapy (59 versus 47 days, *p* = 0.001), with no significant excess of adverse events (20.0% versus 14.6%, *p* = 0.22).

Notably, 44.7% of the cohort would not have met POET eligibility criteria—most often because of an early IV-to-oral switch (<10 days), right-sided involvement, immunosuppression, injection drug use, valvular abscess, or a body mass index ≥ 40 kg/m^2^—yet outcomes in this POET-ineligible subgroup did not differ significantly according to treatment strategy (log-rank *p* = 0.41; adjusted hazard ratio 0.69, 95% CI, 0.28–1.72). An exception concerned patients who underwent an early oral switch (<10 days), among whom treatment failure was numerically higher than with a standard-timing switch (27.8% versus 6.1%, *p* = 0.006), a finding attributed by the authors to small sample size and possible residual indication bias rather than a demonstrated harmful effect of early switching per se. These real-world data suggest that carefully selected patients falling outside the strict POET/ESC framework may still be reasonable candidates for oral step-down therapy, while reinforcing that switching before adequate initial control of infection warrants caution.

### 6.5. Additional Real-World Cohorts, Case Series, and Practice Surveys (2019–2025)

Since the publication of POET, multiple independent cohorts and case series across Europe and North America have examined real-world implementation of oral step-down therapy in IE, complementing the ENDO-ORAL and Danish POETry studies described above. Vroon et al. retrospectively assessed 119 Dutch patients with left-sided IE and found that only 38 (32%) fulfilled all POET eligibility criteria, most commonly failing due to a persistent indication for IV therapy—underscoring how restrictively the original trial criteria apply in unselected populations [36]. In the United States, El-Dalati et al. reported a case series of 32 patients managed by a dedicated multidisciplinary endocarditis team using a standardized oral step-down protocol, with a high rate of clinical success [37]; the same group subsequently published a larger single-center retrospective cohort of 236 patients (93 oral switch, 143 exclusive IV), again finding no significant difference in 90-day relapse (0.7% versus 2.2%), 90-day all-cause mortality (2.8% versus 6.5%), or the composite outcome (3.5% versus 8.6%), including patients with a history of injection drug use [38]. Lundgren et al. described a single-center case series in which only 9 of 136 IE patients underwent oral switch, most often because of poor venous access, patient preference, or IV antibiotic toxicity, highlighting persistently low uptake outside trial settings [39].

Two POET substudies have further refined understanding of oral therapy. Bock et al. performed pharmacokinetic/pharmacodynamic analyses of the oral regimens used in POET and found that the probability of target attainment was high for amoxicillin and linezolid (88–100%) but more variable for moxifloxacin and rifampicin (71–100%), supporting consideration of therapeutic drug monitoring for the latter agents [40]. Bundgaard et al. examined psychological outcomes in POET and found comparable improvements in anxiety and depression scores between the oral and IV groups at 6 months, with numerically lower symptom scores in the oral group, suggesting no psychological disadvantage—and a possible benefit—to earlier discharge [41].

Finally, two surveys conducted after the publication of the 2023 ESC Guidelines confirm that adoption of oral step-down therapy remains limited in everyday practice, corroborating the implementation gap already described in the Danish POETry study. An Emerging Infections Network survey of United States infectious diseases physicians found that, despite trial evidence, 16% never used oral step-down therapy and 53% used it in 10% or fewer of their patients, with only 10% using it in more than a quarter of cases [42]. Similarly, an international survey conducted by the European Society of Clinical Microbiology and Infectious Diseases Study Group for Bloodstream Infections, Endocarditis and Sepsis found substantial heterogeneity in early oral switch practices—influenced by the causative pathogen and the valve involved—with only about half of respondents routinely using this strategy despite guideline endorsement, citing persistent concerns about insufficient evidence [43]. These findings reinforce the importance of educational and structural initiatives.

### 6.6. Systematic Reviews and Meta-Analyses of Oral Step-Down Therapy

Beyond individual cohorts, several systematic reviews and meta-analyses have synthesized the growing body of evidence on oral step-down therapy in IE. An early meta-analysis by Rezar et al., predating most real-world cohorts, found partial oral therapy to be non-inferior to IV therapy in non-critically ill patients with IE [44]. A 2020 Cochrane review by Martí-Carvajal et al. concluded that the overall quality of evidence comparing antibiotic regimens for IE—including route of administration—remained low, highlighting the need for further high-quality trials, a conclusion echoed by the WikiGuidelines consensus statement discussed later [45]. Wald-Dickler et al. subsequently performed a broader systematic review spanning bloodstream infection, osteomyelitis, and IE, concluding that no prospective controlled data support the dogma that intravenous therapy is required for the full treatment duration of these conditions, and that oral step-down is supported by available randomized evidence [46]; the same research group later synthesized the clinical implications of this evidence specifically for bacteremia and endocarditis in a follow-up narrative review [47]. Barda et al. performed a meta-analysis restricted to IE and found no significant differences in microbiological cure, relapse, or mortality between oral switch and continued IV therapy, though the analysis was limited by the small number of eligible studies (six papers, three non-randomized) [48]. Most recently, Mourad et al. conducted a meta-analysis restricted to randomized controlled trials of oral versus IV therapy specifically for *S. aureus* bacteremia or endocarditis and found no difference in treatment failure between routes of administration, while cautioning that the generalizability of these findings to unselected, real-world populations remains uncertain [49]. A related randomized trial in the broader S. *aureus* bacteremia population—the SABATO trial—demonstrated non-inferiority of an early oral switch compared with continued IV therapy in low-risk patients; although not an IE-specific trial, its results, including validation of a lower-dose cotrimoxazole regimen, directly informed the SPILF-AEPEI recommendations discussed later [50].

## 7. Selection of Oral Antibiotic Regimens

For left-sided IE, the best-supported POAT strategy remains a two-agent oral regimen, reflecting the design of the POET trial and the 2023 ESC framework [8,12,17]. However, the necessity of dual therapy versus an appropriately selected single active oral agent remains unresolved, and monotherapy should not be presented as equivalent outside settings supported by specific evidence or expert guidance [51,52]. Pharmacokinetic and pharmacodynamic principles can be used to optimize dosing and maximize antibiotic concentrations at the site of infection. Selected agents must demonstrate high oral bioavailability, ideally exceeding 80% [3]. Both the choice of antimicrobial agents and their dosages are critical determinants of treatment success [Table 2].

For staphylococcal IE, fluoroquinolones should be combined with another active agent, most commonly rifampicin. Among available fluoroquinolones, moxifloxacin appears less prone to the selection of in vivo resistance mutations than ciprofloxacin [29]. TMP-SMX is generally combined with clindamycin or rifampicin [17]. For methicillin-susceptible *S. aureus*, dicloxacillin combined with rifampicin or fusidic acid is an established oral regimen [8]. Dicloxacillin has a moderate and variable oral bioavailability, with an absolute bioavailability of approximately 49% based on serum area-under-the-curve measurements, and is highly protein-bound, with reported binding of approximately 96–97% [53,54]. Its absorption may also be reduced by concomitant food intake. These characteristics may limit and increase the variability of unbound drug exposure. Nevertheless, when administered at high and frequent doses in patients with preserved gastrointestinal absorption and infections caused by highly susceptible isolates, dicloxacillin may achieve adequate pharmacokinetic/pharmacodynamic exposure. Its suitability for oral step-down therapy should therefore be assessed not on bioavailability alone, but also according to isolate-specific MIC, dosing schedule, food administration, gastrointestinal absorption, and the activity of the companion antimicrobial agent. In the POET trial, oral regimens combined two agents selected on the basis of their pharmacokinetic properties and antimicrobial activity, thereby reducing the risk of inadequate treatment if exposure to one component was insufficient [8].

For IE caused by Gram-positive cocci, linezolid represents a potential single-agent oral option, owing to its excellent oral bioavailability (~98%) and broad-spectrum Gram-positive activity. However, rifampicin must not be co-administered with linezolid, as it significantly reduces linezolid serum concentrations through induction of cytochrome P450 enzymes and P-glycoprotein [31,32]. Modern tetracyclines, including doxycycline and minocycline, may also represent reliable alternatives in selected situations [29].

## 8. Practical Considerations and Eligibility Criteria

### 8.1. 2023 ESC Guidelines

The 2023 ESC Guidelines formally endorse POAT in patients with IE caused by *S. aureus*, streptococci, *E. faecalis*, or coagulase-negative staphylococci, within a structured eligibility framework derived largely from the POET trial [12]. These criteria should be verified by the treating physician and, ideally, discussed within a multidisciplinary “Endocarditis Team” including cardiologists, cardiac surgeons, infectious disease specialists, and clinical microbiologists. More recently, complementary guidance from the WikiGuidelines Group consensus statement and the French SPILF-AEPEI position statement has refined, and in some respects broadened, this framework (Section 8.2); the criteria below summarize the ESC framework, while subsequent sections explicitly distinguish these guideline-derived criteria from broader expert guidance and from the authors’ practical synthesis:

- **Infection control criteria:** absence of fever for at least 48 h; C-reactive protein (CRP) level reduced to less than 25% of peak value or below 20 mg/L; leuko-cyte count below 15 × 10^9^/L.
- **Duration of IV therapy:** at least 10 days of IV antibiotic therapy, or at least 7 days following cardiac surgery.
- **No persistent indication for IV therapy:** no evidence of residual valvular abscess, uncontrolled foci of infection, or ongoing bacteremia.
- **Echocardiographic criteria:** transesophageal echocardiography (TEE) demonstrating no indication for cardiac surgery at the time of proposed oral switch.
- **Anthropometric and absorptive criteria:** BMI below 40 kg/m^2^ and no clinical evidence of impaired gastrointestinal absorption.
- **Microbiological criteria:** pathogen susceptibility to available oral agents, with a suitable two-drug oral regimen identifiable based on susceptibility testing.
- **Adherence:** reliable oral intake, absence of cognitive impairment, and high expected treatment compliance.

Following successful oral switch, close clinical follow-up is essential. This should include regular clinical assessment, repeat blood cultures if fever recurs, monitoring of inflammatory markers, and echocardiographic re-evaluation if clinical deterioration occurs. Therapeutic drug monitoring may be considered for agents such as linezolid and, when feasible, for other agents in complex pharmacokinetic situations.

### 8.2. Points of Convergence and Divergence Among International Guidelines and Position Statements

Beyond the 2023 ESC Guidelines, two additional documents refine the framework for oral step-down therapy in IE. The WikiGuidelines Group consensus statement, a crowdsourced evidence appraisal involving 51 experts from 10 countries, concluded that oral transitional therapy is the only aspect of IE management supported by a “clear recommendation,” based on three randomized controlled trials demonstrating that oral switch is at least as effective as continued IV therapy [51]. Rather than prescribing fixed biological or echocardiographic thresholds, this consensus statement proposes a pragmatic, five-point clinical framework for candidacy: clinical stability without an immediate indication for source control or cardiac surgery; bacteremia cleared or clearing without the need for source control; availability of an oral regimen with in-vitro activity supported by published clinical data; reliable gastrointestinal absorption; and absence of socioeconomic determinants of health that would otherwise favor the IV route. It further highlights that the necessity of dual- versus single-agent oral regimens, and the required duration of IV lead-in before switching, remain unresolved, since published studies have used regimens ranging from combination therapy (as in POET) to monotherapy, and IV lead-in periods ranging from none to several weeks [51].

The French SPILF-AEPEI issued a joint position statement in 2025 refining several sections of the 2023 ESC Guidelines considered imprecise by French infectious disease specialists [52]. Regarding oral switch, the SPILF-AEPEI statement largely adopts the POET/ESC stability criteria (apyrexia ≥ 48 h; CRP < 25% of peak value or <20 mg/L; leukocytes < 15 × 10^9^/L; TEE without a residual surgical indication; BMI ≤ 40 kg/m^2^; no malabsorption; adequate psychosocial adherence) but adds practical, country-specific caveats. Because dicloxacillin—the staphylococcal agent used in POET—is unavailable in France, SPILF-AEPEI advises awaiting the results of the ongoing RODEO trial before broadly endorsing oral regimens for staphylococcal IE, while noting that low-dose cotrimoxazole (as validated in the SABATO trial for uncomplicated *S. aureus* bacteremia) may represent a reasonable alternative when rifampicin cannot be used [52]. Moxifloxacin is preferred over other fluoroquinolones given its use in POET and its bactericidal activity against Gram-positive cocci. For streptococcal and enterococcal IE, amoxicillin-based dual therapy (with rifampicin or moxifloxacin) is favored, and amoxicillin monotherapy is considered only pending confirmatory data from RODEO. Unlike the WikiGuidelines statement, SPILF-AEPEI does not recommend routine oral therapy for Gram-negative bacillary IE (HACEK, Enterobacterales, or *Pseudomonas aeruginosa*) outside of individualized, endocarditis-team-guided decisions, citing the rarity of these infections and the paucity of supporting data [52].

Taken together, the ESC, WikiGuidelines, and SPILF-AEPEI documents agree that oral step-down therapy is a reasonable, evidence-supported strategy for selected patients with left-sided streptococcal, enterococcal, and staphylococcal IE, and they converge on broadly similar clinical stability criteria. They differ, however, in the rigidity with which these criteria should be applied: the ESC and SPILF-AEPEI favor a structured, largely quantitative checklist mirroring the POET protocol, whereas the WikiGuidelines statement favors individualized clinical judgment and explicitly acknowledges uncertainty regarding IV lead-in duration and the need for dual therapy. Real-world data, including the Freling et al. and ENDO-ORAL cohorts, suggest that outcomes may remain favorable even when strict trial-derived criteria are not fully met, supporting a more flexible, endocarditis-team-based approach in appropriately selected patients [Table 3].

## 9. Summary Table: Partial Oral Antibiotic Therapy in Infective Endocarditis

**Figure 1:**
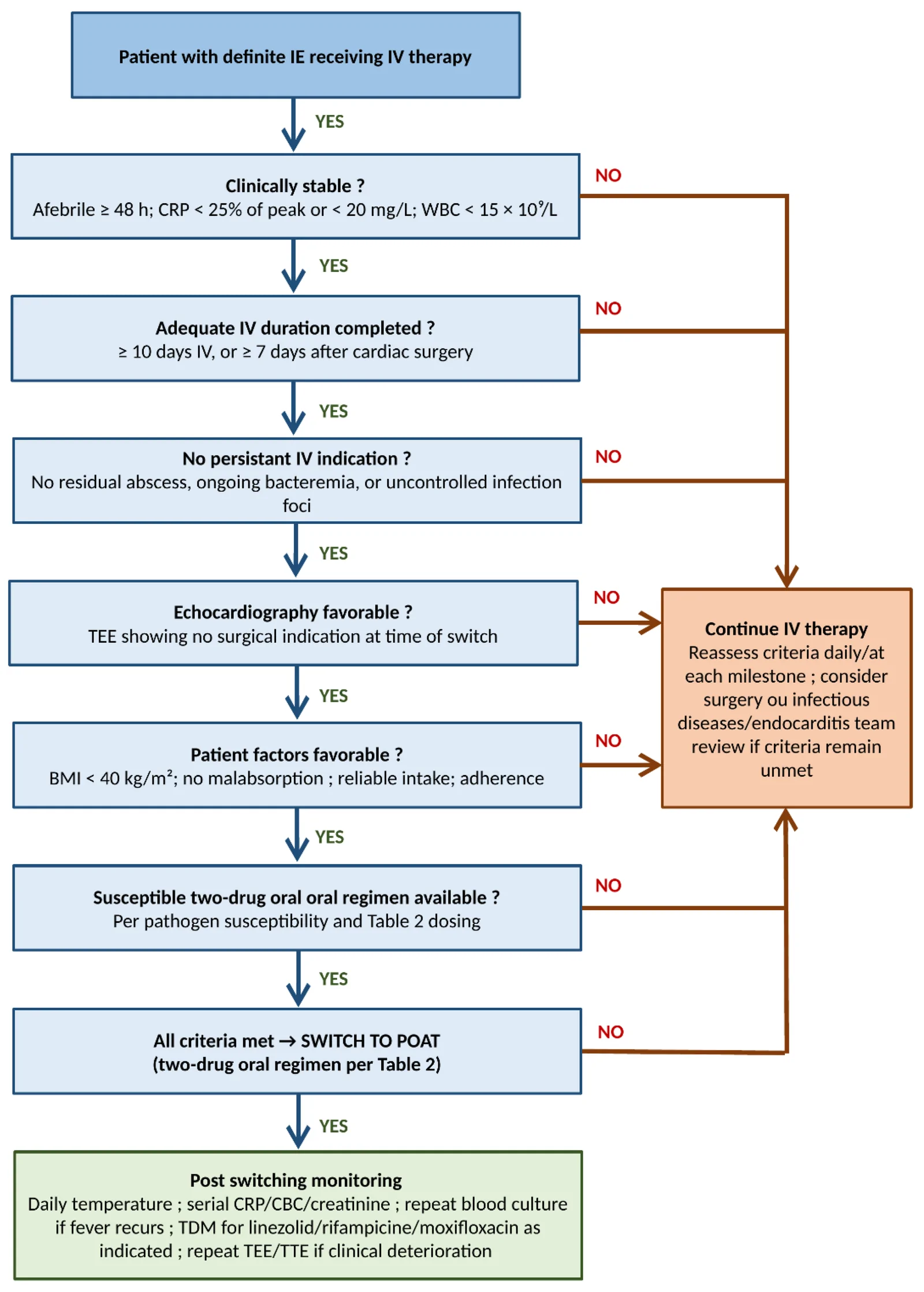
Authors’ proposed practical algorithm for POAT eligibility in IE. This figure represents the authors’ practical synthesis of the 2023 ESC eligibility framework and available evidence and is not a formally validated guideline algorithm. It translates the principal eligibility domains summarized in Table 4 into a sequential bedside checklist, while acknowledging uncertainty regarding the optimal IV lead-in duration, the need for dual-agent oral therapy, and eligibility beyond POET-derived criteria. Patients not meeting a criterion should generally continue IV therapy with reassessment and Endocarditis Team review where appropriate. CRP: C-reactive protein; WBC: white blood cell count; TEE: transesophageal echocardiography; BMI: body mass index; TDM: therapeutic drug monitoring; TTE: transthoracic echocardiography.

Table 4 and Figure 1 provides the authors’ practical synthesis of POAT eligibility, candidate oral regimens, monitoring, and situations favoring continued IV therapy. It integrates trial data and guideline/position-statement recommendations and is intended as a bedside aid rather than a formally validated guideline algorithm.

## 10. Perspectives

The successful implementation of POAT in IE marks a paradigm shift away from a century-old reliance on prolonged IV therapy. However, several important questions remain unanswered, and the field continues to evolve rapidly.

### 10.1. Toward Fully Oral Strategies

The POET trial demonstrated non-inferiority of oral step-down in carefully selected, clinically stable patients after at least 10 days of IV therapy (or at least 7 days after cardiac surgery), and subsequent real-world cohorts have supported this strategy in selected populations. The optimal duration of IV lead-in therapy remains uncertain outside the populations and protocols studied. However, the concept of fully oral therapy—initiation of oral antibiotics from the outset of treatment—remains largely unexplored for IE. By analogy, the OVIVA trial demonstrated non-inferiority of early oral switch (at a median of 7 days) for bone and joint infections [4]. Whether a similar approach could be safely applied to selected low-risk IE patients, particularly those with right-sided disease or streptococcal native valve IE, merits prospective investigation. The ongoing ORAL-IE trial and similar initiatives will hopefully provide clarity on this question.

### 10.2. Right-Sided Infective Endocarditis and People Who Inject Drugs

Data specifically addressing oral therapy for right-sided IE remain limited. Historical evidence supports ciprofloxacin-rifampicin for right-sided staphylococcal endocarditis in injection drug users, an approach further corroborated by small feasibility studies [22,51,52]. Given the challenges of sustained venous access, the high risk of catheter-related complications, and the psychosocial complexity in PWID, this population may represent the group most likely to benefit from earlier or full oral strategies [6,7]. Future trials should specifically address this population and integrate addiction medicine expertise and harm reduction frameworks into treatment protocols.

### 10.3. Prosthetic Valve and Device-Related Endocarditis

Prosthetic valve endocarditis (PVE) and CIED-related IE were not excluded from the POET trial, which enrolled 107 patients with PVE [8]. However, subgroup analyses for PVE remain underpowered, and specific data guiding oral treatment in device-related IE are lacking. Management of CIED-related IE typically requires device extraction in addition to antimicrobial therapy [12,55,56], and the role of POAT in this setting is not yet established. Dedicated observational and interventional studies are warranted.

### 10.4. Patient Monitoring, Telemedicine, and Digital Health

A critical component of safe POAT implementation is appropriate post-discharge monitoring. The transition from inpatient to outpatient management requires robust systems for clinical follow-up, early detection of treatment failure, and rapid access to specialist care. Digital health solutions—including telemedicine consultations, remote monitoring of vital signs, and mobile health applications—represent promising tools to enhance the safety of outpatient oral treatment. Validated standardized pathways for the systematic assessment of POAT candidates and follow-up protocols are needed and represent an active area of development.

### 10.5. Antibiotic Stewardship and Sustainability

POAT can be aligned with antibiotic stewardship principles when used in appropriately selected patients. Oral regimens eliminate the need for central venous catheters, IV formulations, infusion sets, and associated nursing resources, reducing both direct healthcare costs [17] and environmental impact [30]. A shift toward oral therapy reduces the generation of biohazardous plastic waste and decreases the carbon footprint associated with IV drug manufacture and delivery. As healthcare systems increasingly incorporate sustainability into clinical decision-making, POAT may therefore represent a clinically sound and environmentally favorable treatment strategy in selected patients. Its principal stewardship advantages relate to route optimization, avoidance of unnecessary intravascular devices, and resource-efficient care. A direct reduction in the emergence of antimicrobial resistance from POAT itself has not been demonstrated; resistance prevention still depends on pathogen-directed therapy, adequate exposure, appropriate combination therapy when indicated, and avoidance of unnecessarily broad or prolonged treatment.

### 10.6. Optimizing Patient Selection and Personalized Medicine

Currently, eligibility for POAT relies on a combination of clinical, echocardiographic, biological, and microbiological criteria [8,12]. However, these criteria do not fully capture individual pharmacokinetic variability, host immune competence, or genetic factors influencing antibiotic metabolism. Future research may incorporate pharmacogenomics, validated biomarkers of treatment response (beyond CRP), and machine learning–based risk prediction tools to refine patient selection and personalize oral regimen choice [8,17]. The integration of therapeutic drug monitoring into routine POAT protocols is an additional area deserving further evaluation, particularly for agents with narrow therapeutic windows such as linezolid.

### 10.7. Guideline Implementation and Education

Despite randomized evidence and increasing guideline support, POAT remains underutilized in routine clinical practice. The Danish POETry study identified that only 43% of eligible patients received oral step-down therapy, with physician hesitancy being a key barrier [11]. Educational initiatives targeting infectious disease specialists, cardiologists, and internists, as well as the development of standardized institutional POAT protocols and multidisciplinary Endocarditis Team pathways, are critical to bridging the gap between evidence and practice.

### 10.8. Limitations of This Review

This review has several limitations inherent to its narrative format. The literature search was non-systematic and restricted to English-language publications, which may have introduced selection or language bias, and no formal risk-of-bias or study-quality appraisal (e.g., GRADE) was applied to the included studies. Study selection and synthesis reflect the judgment of two authors from two Belgian institutions; while both are practicing infectious diseases specialists, a broader or more geographically diverse author panel might have identified additional perspectives, particularly regarding country-specific antibiotic availability and guideline interpretation. Most of the real-world cohorts discussed are retrospective and subject to residual confounding by indication, notwithstanding the use of propensity-score methods in some (e.g., ENDO-ORAL). Finally, evidence specific to right-sided IE, prosthetic valve and device-related IE, and fully oral strategies from treatment initiation remains sparse, and conclusions in these areas should be considered hypothesis-generating rather than definitive.

## 11. Conclusions

Beyond the established indications of right-sided *S. aureus* IE and endocarditis caused by atypical pathogens, growing evidence supports consideration of POAT in carefully selected patients with IE caused by streptococci, enterococci, and staphylococci. The non-inferiority of POAT has been demonstrated in the POET trial, confirmed by long-term follow-up showing lower all-cause mortality at 5.4 years, and corroborated by real-world implementation studies. The 2023 ESC Guidelines endorse POAT within a structured eligibility framework.

In appropriately selected, clinically stable patients, POAT can reduce hospitalization and avoid catheter-related complications and may reduce healthcare resource use and environmental burden, while randomized evidence supports non-inferior clinical outcomes compared with continued IV therapy in the populations studied. Patient selection, pathogen susceptibility, reliable oral drug exposure, and close clinical monitoring remain central to safe implementation. The POET-derived two-drug strategy is the best-established approach, but the optimal duration of IV lead-in therapy and the role of oral monotherapy remain incompletely defined.

Evidence remains limited for right-sided IE, fully oral strategies from treatment initiation, and device-related IE. Future clinical trials and real-world implementation studies will be instrumental in further defining the scope of oral antibiotic therapy in the management of infective endocarditis. Emerging real-world data, such as the ENDO-ORAL study, together with complementary international guidance from the WikiGuidelines Group and SPILF-AEPEI, suggest that selected patients outside strict POET-derived criteria may also have favorable outcomes with oral step-down therapy—an area that warrants confirmation in dedicated prospective trials such as RODEO and OraPAT-IE GAMES [35,51,55,57,58].

## Figures and Tables

**Table 1:** Oral antibiotic regimens used in the POET trial according to the causative microorganism (Iversen et al., 2019) [8].

Microorganism	Oral Antibiotic Regimen(s)
Penicillin- and methicillin-sensitive *S. aureus*/CoNS	Amoxicillin + fusidic acid
	Amoxicillin + rifampicin
	Linezolid + fusidic acid
	Linezolid + rifampicin *
Methicillin-sensitive *S. aureus*/CoNS	Dicloxacillin + fusidic acid
	Dicloxacillin + rifampicin
	Linezolid + fusidic acid
	Linezolid + rifampicin *
Methicillin-resistant CoNS	Linezolid + fusidic acid
	Linezolid + rifampicin *
*Enterococcus faecalis*	Amoxicillin + rifampicin
	Amoxicillin + moxifloxacin
	Linezolid + rifampicin *
	Linezolid + moxifloxacin
Streptococci (MIC penicillin < 1 mg/L)	Amoxicillin + rifampicin
	Linezolid + rifampicin *
	Linezolid + moxifloxacin
Streptococci (MIC penicillin ≥ 1 mg/L)	Linezolid + rifampicin *
	Moxifloxacin + rifampicin
	Moxifloxacin + clindamycin

CoNS: coagulase-negative staphylococci; MIC: minimum inhibitory concentration. * Although linezolid–rifampicin combinations were used in the POET trial, subsequent pharmacokinetic data have demonstrated a clinically relevant reduction in linezolid exposure with rifampicin co-administration; this combination is therefore currently discouraged [31,32].

**Table 2:** Oral antimicrobial agents and candidate dosing regimens for partial oral antibiotic therapy in infective endocarditis.

Antibiotic Agent	Oral Dosage	Main Indications and Comments	References
Amoxicillin	1 g four times daily	Streptococcal IE; enterococcal IE in selected combination regimens.	[8,17,26,52]
Dicloxacillin	1 g four times daily	MSSA/CoNS IE; POET used in two-drug regimens. Variable oral exposure; administer with attention to food and PK factors.	[8,52,53,54]
Moxifloxacin	400 mg once daily	Selected streptococcal, enterococcal, or staphylococcal IE regimens; used in POET.	[8,29,52]
Levofloxacin	750 mg–1 g once daily	Alternative fluoroquinolone in selected regimens; use should be susceptibility- and guidance-based.	[17,51]
TMP-SMX	960/4800 mg daily (in divided doses)	MSSA IE; evidence includes high-dose TMP-SMX plus clindamycin. Lower-dose cotrimoxazole strategies remain context-specific.	[25,50,52]
Linezolid	600 mg twice daily	Gram-positive IE option with near-complete oral bioavailability; monitor hematologic/neurologic toxicity. Avoid rifampicin co-administration.	[17,23,24,31,32]
Rifampicin	600 mg once or twice daily	Combination agent; major drug interactions. Do not combine with linezolid because rifampicin lowers linezolid exposure.	[8,17,31,32]
Clindamycin	600 mg three times daily	Combination agent used with TMP-SMX in selected *S. aureus* regimens.	[25]
Fusidic acid	750 mg twice daily	Combination agent in selected staphylococcal regimens; avoid monotherapy because resistance emerges rapidly.	[8,17]

TMP-SMX: trimethoprim–sulfamethoxazole; Note: dicloxacillin is not commercially available in several countries, including France, where alternative regimens (e.g., cotrimoxazole-based) may be required. Dose adjustment for renal impairment is required for amoxicillin, TMP-SMX, and clindamycin (per local formulary guidance), and hepatic function should be monitored during rifampicin, fusidic acid, or linezolid therapy; dicloxacillin, moxifloxacin, and levofloxacin dosing should also be reviewed in patients with significant renal impairment.

**Table 3:** Comparison of guidance on partial oral antibiotic therapy for infective endocarditis across the 2023 ESC Guidelines, the 2023 WikiGuidelines Group consensus statement, and the 2025 SPILF-AEPEI position statement.

Domain	ESC 2023 Guidelines (Delgado et al.) [12]	WikiGuidelines 2023 (McDonald et al.) [51]	SPILF-AEPEI 2025 (Le Moing et al.) [52]
Overall recommendation	Formal endorsement, strict multi-criteria checklist	“Clear recommendation” that oral switch is at least as effective as IV; individualized selection	Endorses ESC/POET framework with French-specific refinements
IV lead-in duration	≥10 days, or ≥7 days after cardiac surgery	Unresolved; published studies range from no lead-in to several weeks	≥10 days, or ≥7 days after valve surgery (as in POET)
Mono- vs. dual-agent oral regimen	Two-drug combination preferred	Unresolved; monotherapy reported effective in several series	Dual therapy per POET; monotherapy (e.g., amoxicillin alone) pending RODEO results
Staphylococcal IE oral options	Dicloxacillin + rifampicin/fusidic acid; linezolid-based regimens	Dicloxacillin, TMP-SMX, linezolid, or fluoroquinolone + rifampicin	Awaiting RODEO trial; dicloxacillin unavailable in France; low-dose cotrimoxazole a possible alternative if rifampicin unusable
Preferred fluoroquinolone	Moxifloxacin	Levofloxacin or moxifloxacin	Moxifloxacin
Gram-negative (HACEK, Enterobacterales) IE	Not specifically addressed for oral switch	Oral fluoroquinolone reasonable once stabilized	Not routinely recommended outside individualized, endocarditis-team-guided decisions
Key eligibility thresholds	Afebrile ≥ 48 h; CRP < 25% of peak or <20 mg/L; WBC < 15 × 10^9^/L; TEE without surgical indication; BMI < 40 kg/m^2^; reliable adherence	Clinical stability; bacteremia cleared/clearing; susceptible oral regimen available; reliable GI absorption; no socioeconomic barrier to oral route	Same quantitative criteria as ESC/POET

**Table 4:** Authors’ practical synthesis of POAT in IE: eligibility criteria, antibiotic regimens, monitoring, and contraindications.

**A. Eligibility Criteria (2023 ESC Guidelines)**		**References**
Clinical stability	Afebrile ≥ 48 h; CRP < 25% of peak or <20 mg/L; WBC < 15 × 10^9^/L	[8,12,51,52]
Duration of IV therapy	≥10 days IV, or ≥7 days after cardiac surgery	[8,12,51,52]
No persistent IV indication	No residual abscess, ongoing bacteremia, or uncontrolled infection foci	[8,12,51,52]
Echocardiography	TEE showing no surgical indication at time of switch	[8,12,52]
Patient factors	BMI < 40 kg/m^2^; no impaired gastrointestinal absorption; reliable oral intake; adequate treatment adherence	[8,12,51,52]
Eligible pathogens	Streptococci, *E. faecalis*, *S. aureus* (methicillin-susceptible or -resistant), coagulase-negative staphylococci	[8,12,51,52]
**B. Candidate Oral Regimens by Pathogen (POET/ESC-Based Combinations; Alternatives Remain Context-Dependent)**		**References**
Streptococci (penicillin MIC < 0.12 μg/mL)	Amoxicillin 1 g QID + rifampicin 600 mg OD/BD or + moxifloxacin 400 mg OD; Moxifloxacin 400 mg OD + clindamycin 600 mg TID	[8,17,51,52]
Streptococci (penicillin MIC ≥ 1 mg/L)	Linezolid 600 mg BD + moxifloxacin 400 mg OD; Moxifloxacin 400 mg OD + rifampicin 600 mg OD/BD, Moxifloxacin 400 mg OD + clindamycin 600 mg TID	[8,17,51,52]
*E. faecalis*	Amoxicillin 1 g QID + rifampicin 600 mg OD/BD; Amoxicillin 1 g QID + moxifloxacin 400 mg OD	[8,17,51,52]
MSSA/methicillin-susceptible CoNS	Dicloxacillin 1 g QID + rifampicin 600 mg OD/BD; Dicloxacillin 1 g QID + fusidic acid 750 mg BD; Linezolid 600 mg BD + fusidic acid 750 mg BD; TMP-SMX 960 mg BD + clindamycin 600 mg TID or + rifampicin 600 mg OD/BD	[8,17,25,50,52]
MRSA/methicillin-resistant CoNS	Linezolid 600 mg BD + fusidic acid 750 mg BD; Linezolid 600 mg BD + moxifloxacin 400 mg OD;	[8,17,51,52]
Atypical pathogens (Brucella, Coxiella, Bartonella, *Tropheryma whipplei*)	Doxycycline-based regimens (with TMP-SMX, rifampicin, or hydroxychloroquine depending on pathogen and clinical context)—already standard of care	[1,2,12,20]
**C. Monitoring During POAT**		**References**
Clinical	Daily temperature monitoring; assessment of new symptoms (dyspnea, neurological changes, chest pain); reassessment within 24 h if fever recurs	[8,12,40,51,52]
Biological	Serial CRP, CBC, creatinine, hepatic enzymes; repeat blood cultures if fever recurs or clinical deterioration	[8,12,40]
Therapeutic drug monitoring (TDM)	Consider TDM for linezolid (risk of toxicity), rifampicin (low serum levels possible), moxifloxacin; mandatory if subtherapeutic levels suspected or toxicity occurs	[8,12,51,52]
Echocardiography	Repeat TEE or TTE if clinical deterioration, recurrent fever, or suspicion of new surgical complication	[8,12,51,52]
**D. Situations Generally Favoring Continued IV Therapy**		**References**
Strong reasons to continue IV therapy	Hemodynamic instability or septic shock; ongoing bacteremia or uncontrolled infection; active valvular abscess or perivalvular extension; active surgical indication; BMI ≥ 40 kg/m^2^ or documented malabsorption; no available oral regimen active against the pathogen	[8,12,51,52]
High-risk situations (relative)	MRSA IE (limited oral regimen options); CIED-related IE (typically require device extraction + IV therapy); fungal endocarditis; IE in immunocompromised host; prosthetic valve IE without surgical stabilization	[12,51,52]
Drug-specific contraindications	Rifampicin + linezolid combination (pharmacokinetic interaction); fluoroquinolones in patients with QTc prolongation or at risk of tendinopathy; linezolid in patients on serotonergic drugs	[17,31,32]

CRP: C-reactive protein; WBC: white blood cell count; TEE: transesophageal echocardiography; TTE: transthoracic echocardiography; MIC: minimum inhibitory concentration; MSSA: methicillin-susceptible *S. aureus*; MRSA: methicillin-resistant *S. aureus*; CoNS: coagulase-negative staphylococci; CIED: cardiac implantable electronic device; TMP-SMX: trimethoprim–sulfamethoxazole; QID: four times daily; BD: twice daily; TID: three times daily; OD: once daily; TDM: therapeutic drug monitoring. Linezolid should not be combined with rifampicin because rifampicin significantly reduces linezolid exposure [31,32]. Although this combination was used in the POET trial, it is not included among the candidate regimens proposed in this practical synthesis.

## Data Availability

No new date were created or analyzed in this study. Data sharing is not applicable to this article.

## Abbreviations

The following abbreviations are used in this manuscript:

IE	Infective endocarditis
IV	Intravenous
POAT	Partial oral antibiotic therapy
POET	Partial Oral Treatment of Endocarditis
SPILF	Société de Pathologie Infectieuse de Langue Française (https://www.infectiologie.com/)
AEPEI	Association pour l’Étude et la Prévention de l’Endocardite Infectieuse
CIED	Cardiac implantable electronic device
ESC	European Society of Cardiology
AHA	American Heart Association
TMP-SMX	Trimethoprim-sulfamethoxazole
MSSA	Methicillin-susceptible *Staphylococcus aureus*
MRSA	Methicillin-resistant *Staphylococcus aureus*
MIC	Minimum inhibitory concentration
PWID	People who inject drugs
CoNS	Coagulase-negative staphylococci
NVE	Native valve endocarditis
PVE	Prosthetic valve endocarditis
TTE	Transthoracic echocardiography
TEE	Transesophageal echocardiography
CRP	C-reactive protein
